# Implementing Culturally Tailored Diabetes Prevention in Hispanic Communities: An Integrative Review Using the RE-AIM Framework

**DOI:** 10.5888/pcd23.260050

**Published:** 2026-10-29

**Authors:** Rachell Nguyen

**Affiliations:** 1 Texas Woman’s University College of Nursing, Houston, Texas

## Abstract

**Introduction:**

Hispanic adults in the US have disproportionately high rates of diabetes and face persistent barriers to accessing evidence-based prevention programs. Despite the demonstrated efficacy of culturally tailored diabetes prevention interventions, translation into routine community practice is markedly limited. This integrative review examined implementation processes, barriers, and facilitators of culturally tailored diabetes prevention interventions in Hispanic communities, using the Reach, Effectiveness, Adoption, Implementation, Maintenance (RE-AIM) framework to identify strategies that support population-level impact.

**Methods:**

Comprehensive searches of 5 databases (PubMed, CINAHL, Web of Science, EMBASE, ScienceDirect) from 2005 through 2025 identified 69 sources; 37 (empirical studies, study protocols, and reviews) reporting implementation data were analyzed. Data extraction and synthesis aligned with the RE-AIM framework and used constant comparative methods. Quality appraisal used the Johns Hopkins Evidence-Based Practice tool. This review was not registered, consistent with integrative review methods.

**Results:**

Implementation facilitators included cultural concordance, community health worker models, community-based delivery, and family-centered programming. Barriers included funding instability, workforce shortages, male underrepresentation, and limited maintenance data. Interventions achieved clinically meaningful weight loss (3.2 kg–5.8 kg), but real-world outcomes were smaller than those reported in efficacy trials. Community-based organizations were the primary adopters, although resource constraints were common. Only 18% of studies reported outcomes beyond 12 months, limiting the assessment of sustained impact.

**Conclusion:**

Culturally tailored diabetes prevention interventions are effective and acceptable in Hispanic communities; however, implementation gaps in adoption, scaling ability, and sustainability limit population-level impact. Successful translation requires cultural concordance, community partnerships, flexible delivery models, and workforce development. Addressing structural barriers through policy support for community health worker integration, sustainable funding mechanisms, and health care–community partnerships is essential to achieving equitable diabetes prevention at scale.

**Summary**

**What is known on this topic?**

Culturally tailored diabetes prevention interventions are effective at reducing diabetes risk among Hispanic adults; however, their translation into routine community practice is limited, despite the population’s high diabetes prevalence.

**What is added by this report?**

This Reach, Effectiveness, Adoption, Implementation, Maintenance (RE-AIM) framework analysis of 37 studies identified implementation gaps in adoption, sustainability, and male engagement, while documenting successful strategies including community health worker models, community-based delivery, and family-centered programming.

**What are the implications for public health practice?**

Achieving equitable diabetes prevention requires policy support for community health worker integration, sustainable funding mechanisms beyond time-limited grants, workforce development that addresses bilingual and bicultural shortages, and health care–community partnerships.

## Introduction

Hispanic adults are the largest ethnic minority group in the US and have a disproportionately high prevalence of type 2 diabetes, nearly 50% higher than that of non-Hispanic White adults, with increased risk and earlier onset of complications, including retinopathy, nephropathy, and cardiovascular disease (1). Despite evidence supporting culturally tailored diabetes prevention interventions, translation into routine community practice is limited (2).

The persistent gap between “what works” in controlled research settings and “what gets implemented” in real-world practice is a barrier to reducing health disparities. Although the National Diabetes Prevention Program (NDPP) has demonstrated efficacy, data show that only 35.5% of participants complete core requirements, with even lower rates among Hispanic participants (3). Programs are limited in Hispanic communities, particularly in rural areas, along with retention challenges and cultural–linguistic barriers that undermine effectiveness (4,5).

Although African American and Asian American communities also have high diabetes risk and face culturally specific barriers to program implementation, each warranting dedicated investigation, the Hispanic population is uniquely underserved by existing NDPP infrastructure, with persistently lower enrollment and completion rates than other high-risk groups (3). The cultural, linguistic, and structural heterogeneity in Hispanic communities, including variation by country of origin, acculturation level, immigration status, and geographic distribution, requires a population-focused implementation science lens.

Implementation science provides frameworks for implementing, adapting, sustaining, and scaling evidence-based interventions across diverse real-world contexts (6). The Reach, Effectiveness, Adoption, Implementation, and Maintenance (RE-AIM) framework explicitly addresses implementation dimensions that are overlooked in traditional efficacy research (7). RE-AIM examines 5 interconnected dimensions: reach (proportion and representativeness of participants), effectiveness (impact in real-world settings), adoption (organizational uptake), implementation (fidelity, adaptation, and delivery consistency), and maintenance (sustained effects and organizational continuation).

Although previous reviews examined the effectiveness of culturally tailored diabetes prevention interventions (8), they offered limited guidance on implementation processes, organizational adoption patterns, strategies for balancing fidelity and adaptation, workforce constraints, and factors predicting sustainability.

Health care and public health workforces, including nurses, community health workers, and health educators, play a central role in translating evidence-based prevention into practice; however, implementation strategies that support their success are poorly characterized. This integrative review addresses these gaps by synthesizing implementation science evidence on culturally tailored diabetes prevention for Hispanic adults using the RE-AIM framework. This research sought to answer the following questions:

1. What implementation facilitators and barriers affect culturally tailored diabetes prevention interventions for Hispanic adults across RE-AIM domains?
2. What evidence-based strategies support the successful adoption, delivery, and sustainability of culturally tailored diabetes prevention in Hispanic communities?

## Methods

### Design

This integrative review used Whittemore and Knafl’s (9) 5-stage methodology, allowing inclusion of diverse study designs to provide a comprehensive understanding of implementation phenomena. The framework guided data extraction and synthesis across 5 implementation domains. The review followed PRISMA (Preferred Reporting Items for Systematic Reviews and Meta-Analyses) 2020 reporting standards for systematic reviews.

### Search strategy

Comprehensive searches were conducted in PubMed, CINAHL, Web of Science, EMBASE, and ScienceDirect from January 2005 through December 2025. Search strategies combined 4 concept domains: diabetes prevention and prediabetes; Hispanic, Latino, Latina, and Latinx populations; cultural adaptation and tailoring; and implementation science constructs (ie, implementation, dissemination, adoption, scalability, feasibility, acceptability, sustainability, barriers and facilitators, community-based participatory research [CBPR], and fidelity).

After removing irrelevant articles, 320 records were obtained; this decreased to 296 unique records after removing 24 duplicates (Figure). A full-text review of 296 articles identified 69 sources that met the initial inclusion criteria. Because only sources reporting implementation data relevant to at least 1 RE-AIM domain were retained for the main analysis, this number was reduced to 37 (1,2,4–6,8,10–40) and organized in Zotero reference manager. Consistent with integrative review methodology (9), the 37 sources include empirical studies, study protocols with implementation detail, and reviews; some contributed data to multiple domains and are therefore represented as separate analytic units.

**Figure:**
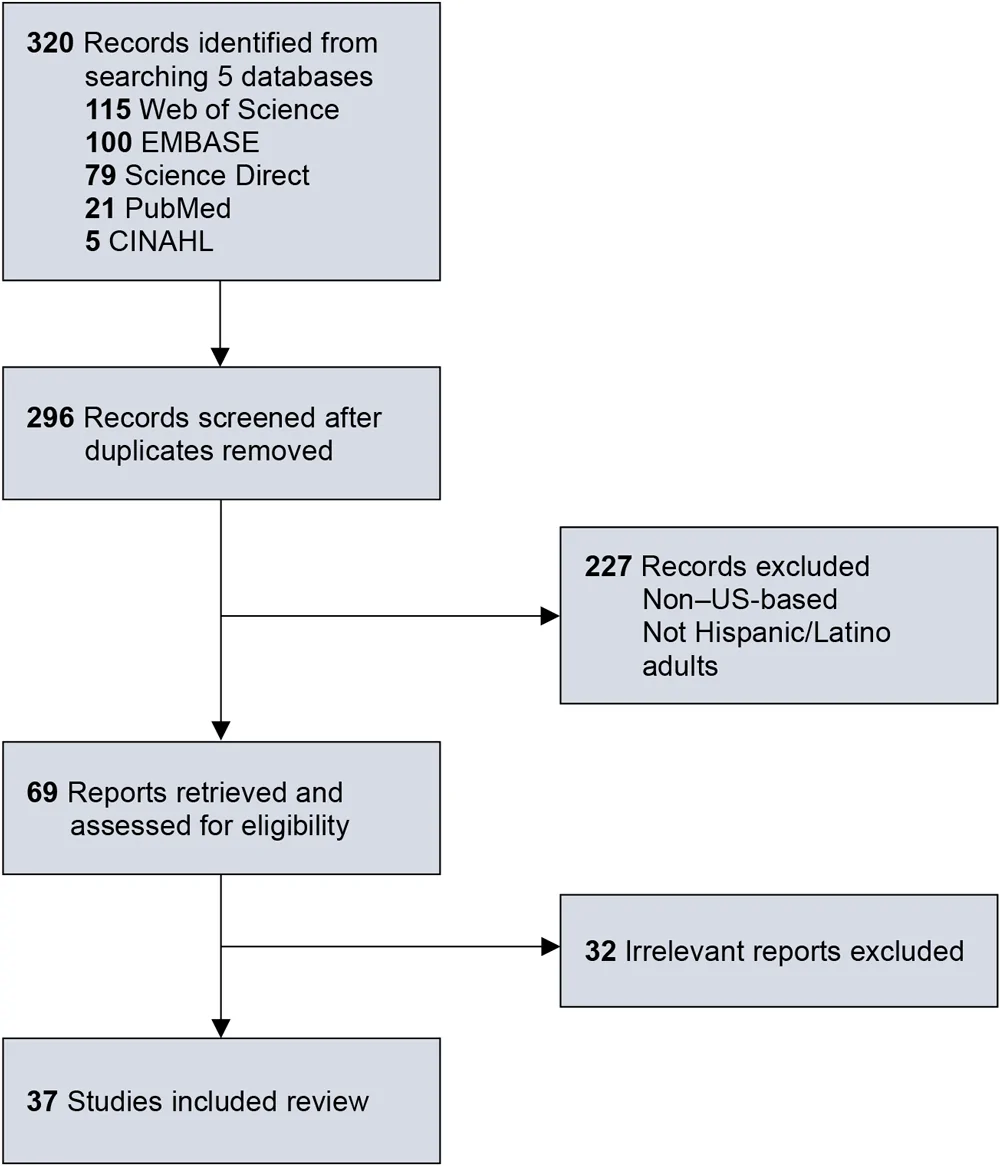
PRISMA (Preferred Reporting Items for Systematic Reviews and Meta-Analyses) 2020 flow diagram for systematic literature search and study selection process.

All screening, data extraction, and quality appraisal were conducted by a single reviewer (R.N.) with expertise in diabetes management and implementation science, using standardized tools and procedures to minimize bias. Title and abstract screening were conducted systematically using predetermined inclusion criteria. Full-text review applied explicit decision rules documented in an extraction protocol. Detailed characteristics of all 37 included studies, including design, population, intervention type, cultural adaptation strategy, and key findings by RE-AIM domain, were tabulated. 

### Sample

Inclusion criteria were peer-reviewed sources that were published in English between 2005 and 2025, were conducted in the US, involved Hispanic/Latino adults (aged ≥18 y), addressed diabetes prevention interventions, reported cultural adaptation components, and reported data on 1 or more RE-AIM domains. Consistent with integrative review methodology, which permits synthesis of empirical and nonempirical literature (9), both primary sources (empirical studies) and secondary sources (study protocols, reviews, and program summaries) were eligible when they described implementation processes, barriers, or facilitators. Study designs included randomized controlled trials (RCTs), quasi-experimental studies, mixed-methods studies, qualitative studies, program evaluations, CBPR studies, and feasibility or pilot studies when reporting implementation outcomes. Early phase feasibility and proof-of-concept studies were retained when they reported implementation processes relevant to domains, despite limited outcome data.

Exclusion criteria were sources with no implementation content (including reviews, commentaries, and protocols that described only intervention efficacy), studies on pediatric populations, studies with no cultural adaptation, efficacy trials without implementation context, and non–peer-reviewed literature.

### Measurements

All studies meeting initial inclusion criteria were appraised by using the Johns Hopkins Evidence-Based Practice appraisal tool, which evaluates the level and quality of evidence. Of the 69 sources screened, 37 were retained and rated level I through level III, with high (A) or good (B) methodological quality; these 37 sources formed the basis for the results synthesis. Empirical studies were appraised on their design; protocols, reviews, and summaries were appraised as level III nonexperimental evidence on the basis of the implementation detail they provided. Lower-quality studies that provided unique contextual or implementation insights were retained but weighted accordingly in interpretation. Standardized forms captured study characteristics, population characteristics, intervention characteristics, cultural adaptation strategies, and implementation data for each domain. Many studies provided data relevant to multiple domains; the primary domain each study explicitly addressed was documented, while the results synthesis incorporated all relevant data across domains.

### Analytic strategy

Data analysis used constant comparative methods and thematic synthesis. The initial analysis organized findings from the 37 sources by domain. Within each domain, iterative coding identified patterns and themes. Implementation barriers and facilitators were categorized by using multilevel socioecological frameworks. Given substantial heterogeneity, narrative synthesis rather than meta-analysis was used.

## Results

### Study characteristics

Thirty-seven sources published from 2005 through 2025 informed the synthesis: 24 empirical studies (RCTs, quasi-experimental and pilot studies, and qualitative, observational, and mixed-methods studies), 6 study protocols with implementation detail, and 7 reviews or program summaries (Table 1). Sample sizes ranged from 21 to 143,000 participants, reflecting both intensive community-based feasibility studies and large-scale national program evaluations. Studies were geographically concentrated in regions with substantial Hispanic populations, particularly the Southwest (California, Texas, Arizona, New Mexico), with additional representation from Florida, Illinois, New York, and North Carolina. Approximately 44% of studies explicitly referenced implementation science frameworks or constructs. Methodological quality, assessed using the Johns Hopkins Evidence-Based Practice criteria, were rated either high or good (Table 2). All 37 studies contributed meaningfully to understanding the implementation of culturally tailored diabetes prevention across diverse Hispanic populations and settings.

**Table 1:** Methodological Quality Appraisal of Studies (N = 37) Assessed by Using the JHNEBP Tool^a^ in an Integrative Review on Implementation Processes, Barriers, and Facilitators of Culturally Tailored Diabetes Prevention Interventions in Hispanic Communities, US, 2005–2025

Study author (year)	Study design	Sample/setting	RE-AIM domain(s) informed	JHNEBP level^b^	Quality (A–C)^c^	Key strengths	Key limitations
Ariel-Donges et al (10), 2020	Observational	Rural/urban NDPP	Reach	III	B	Access disparities	Descriptive
Balan et al (11), 2025	Program evaluation	Hispanic women	Adoption	V	B	Equity emphasis	Executive summary
Brown et al (12), 2018	Qualitative	Mexican American adults	Implementation	III	B	Rich participant voice	No outcomes
Burd et al (2), 2020	Policy implementation	Medicare NDPP	Adoption	III	B	System-level insight	No individual outcomes
Cranston et al (13), 2025	Community evaluation	Inclusive DPP	Implementation	III	B	Equity-driven	Localized
Dineen et al (6), 2022	Qualitative	Community sites	Implementation	III	B	Implementation depth	No outcomes
Fontil et al (14), 2016	Pilot feasibility	Low-income Hispanic adults	Implementation	II	B	Digital adaptation	Short follow-up
Franklin et al (15), 2025	Study protocol	Hispanic adults	Implementation	NA	NA	Family-centered	Early phase
Gutierrez Chavez et al (4), 2025	Qualitative	NDPP coaches	Implementation	III	B	Cultural delivery	Small sample
Horowitz et al (16), 2011	Mixed methods	Community-based intervention	Reach, implementation	III	B	Strong CBPR approach	Limited control
Johnston et al (5), 2025	Scoping review	NDPP reach	Reach	V	B	Comprehensive	Secondary data
Joiner et al (17), 2025	Qualitative	Hispanic adults	Reach	III	B	Awareness focus	No intervention
Joseph (1), 2023	Narrative review	Minority populations	Adoption	V	B	Equity framing	Broad scope
Kim et al (18), 2018	Study protocol	Medicaid population	Implementation	NA	NA	Real-world context	Selection bias
Koh (19), 2023	Integrative review	Hispanic adults	Effectiveness	V	B	Nutrition focus	Dietary self-report
Ling et al (20), 2023	Narrative review	Minority adults	Implementation	V	B	Peer support	Limited Hispanic-only data
Loya et al (21), 2025	Pilot feasibility	Hispanic adults	Effectiveness	II	B	Physical activity focus	Small sample
McCurley et al (22), 2017	Pilot trial	Latina women	Effectiveness	II	B	Cultural relevance	Small sample
McCurley et al (8), 2017	Systematic review	Hispanic adults	Effectiveness	II	A	Strong synthesis	Not implementation-focused
McEwen et al (23), 2010	Intervention trial	Border populations	Effectiveness	II	B	Social support	Older model
Meulenberg et al (24), 2025	Survey	NDPP participants	Adoption	III	B	Barrier analysis	Self-report
Miranda et al (25), 2025	Qualitative	Mexican-origin men	Reach	III	B	Cultural narratives	Gender-specific
Ockene et al (26), 2012	RCT	Latino community adults	Effectiveness	I	A	Randomization, objective outcomes	Single region
Patel et al (27), 2025	Scoping review	Population level	Adoption	V	A	Multilevel lens	Heterogeneous data
Risica et al (28), 2018	Study protocol	Latino adults	Implementation	NA	NA	Program rationale	Outcomes pending
Rosas et al (29), 2016	Study protocol: RCT	Primary care Latino adults	Effectiveness	NA	NA	Rigorous design	Clinic-based only
Rosas et al (30), 2018	Study protocol: RCT	Latino men	Effectiveness	NA	NA	Male engagement	Gender-specific
Rosas et al (31), 2018	Program description	Latino adults	Adoption	V	B	Patient engagement	Limited follow-up
Rosas et al (32), 2024	Study protocol: RCT	Latina women	Effectiveness	NA	NA	Innovative groceries	Resource intensive
Sanchez et al (33), 2022	Qualitative	Latinx families	Reach	III	B	Family dynamics	Regional
Saju et al (34), 2022	Qualitative	Diverse adults	Reach	III	B	Equity focus	Recall bias
Soltero et al (2019) (35), 2019	Multilevel intervention	Community settings	Adoption	I	A	Multisector model	Complex replication
Vincent et al (36), 2007	Feasibility	Mexican American adults	Implementation	II	B	Early cultural tailoring	Dated context
Vincent et al (2013) (37), 2013	Mixed methods	Mexican-descent adults	Reach, implementation	III	B	Recruitment/retention focus	Small sample
Vincent et al (38), 2014	Quasi-experimental	Community based	Effectiveness	II	B	Cultural tailoring	No randomization
Joachim-Célestin et al (39), 2020	Qualitative	Latina participants	Reach, implementation	III	B	Cultural insight	Subjective data
Lewis et al (40), 2025	Longitudinal qualitative	Vida Sana	Maintenance	III	B	Sustainability insight	Qualitative only

Abbreviations: DPP, diabetes prevention program; JHNEBP, Johns Hopkins Evidence-Based Practice; NA, not applicable; NDPP, National Diabetes Prevention Program; RCT, randomized controlled trial; RE-AIM, Reach, Effectiveness, Adoption, Implementation, and Maintenance.

a This table summarizes the methodological quality appraisal of the 37 sources (empirical studies, study protocols, and reviews) included in the integrative review, using the JHNEBP appraisal tool.

b Level I, experimental/RCT; Level II, quasi-experimental; Level III, nonexperimental, qualitative, mixed methods.

c A indicates high quality, B indicates good quality, and C indicates low quality.

**Table 2:** Characteristics of Studies (N = 37) in an Integrative Review on Implementation Processes, Barriers, and Facilitators of Culturally Tailored Diabetes Prevention Interventions in Hispanic Communities, US, 2005–2025

Study author (year)	Study design	Sample population	Setting	Intervention type	Cultural adaptation strategy	Analytic approach	Key barriers	Key facilitators	RE-AIM domain(s)
Ariel-Donges et al (10), 2020	Observational	Rural/urban NDPP participants	Rural and urban US communities	NDPP access comparison	Geographic access analysis	Descriptive/comparative analysis	Fewer rural sites; transportation; geographic distance	Identification of access disparities to inform targeted outreach	Reach
Balan et al (11), 2025	Program evaluation	Hispanic women	Community/health system settings	Diabetes prevention program evaluation	Equity-focused program design for Black and Hispanic women	Executive summary/synthesis of lessons learned	Resource constraints; need for tailored strategies	Equity-driven program design; actionable lessons learned	Adoption
Brown et al (12), 2018	Qualitative	Mexican American adults (Starr County)	Border community (Starr County, TX)	Diabetes prevention focus groups	Community-based, border-region cultural framing	Thematic/qualitative analysis of focus groups	Limited program availability; no quantitative outcomes	Rich participant voice on cultural perceptions and needs	Implementation
Burd et al (2), 2020	Policy implementation	Medicare NDPP beneficiaries (national)	Medicare program/national health system	Medicare expansion of NDPP lifestyle intervention	National program adaptation for diverse populations	Policy/translational analysis	No individual-level outcomes; system-level barriers to uptake	System-level insight; reimbursement pathway model	Adoption
Cranston et al (13), 2025	Community evaluation	Inclusive DPP participants	Community-based DPP sites	Inclusive DPP	Equity-driven program adaptations for inclusion	Program evaluation/descriptive analysis	Localized findings; limited generalizability	Equity-driven approach to inclusive program design	Implementation
Dineen et al (6), 2022	Qualitative	Community site staff/participants	2 Community-based DPP sites	DPP implementation	Community site–based delivery adaptation	Qualitative implementation analysis	No outcome data; site-specific findings	Implementation depth; site-level insights	Implementation
Fontil et al (14), 2016	Pilot feasibility	Low-income Hispanic adults	Safety-net hospital system + digital health partner	Digital health diabetes prevention program	Digital adaptation for low-income, Spanish-speaking patients	Feasibility analysis	Short follow-up; digital literacy gaps	Digital adaptation feasible in safety-net setting	Implementation
Franklin et al (15), 2025	Study protocol	Hispanic/Latino adults at high risk for type 2 diabetes	Community/family setting	PrevenganT2 family-based diabetes prevention intervention	Family-based, culturally responsive program design	Proof-of-concept/feasibility analysis	Early-phase program; limited outcome data	Family-centered design promotes engagement	Implementation
Gutierrez Chavez et al (4), 2025	Qualitative	NDPP coaches	NDPP delivery sites	NDPP delivery (coach perspective)	Culturally responsive coaching strategies	Qualitative thematic analysis	Small sample size	Cultural delivery strategies identified by coaches	Implementation
Horowitz et al (16), 2011	Mixed methods	Community-based intervention participants	Historically underserved urban community	Community-based diabetes prevention translation model	CBPR-driven community partnership model	Mixed methods (quantitative outcomes + qualitative process)	Limited experimental control	Strong CBPR approach; successful translation model	Reach, Implementation
Johnston et al (5), 2025	Scoping review	NDPP participants (secondary data)	NDPP sites	NDPP reach analysis	Secondary analysis of reach across populations	Secondary/observational data analysis	Secondary data limitations	Comprehensive national reach data	Reach
Joiner et al (17), 2025	Qualitative	Hispanic adults with prediabetes risk	Community settings	Prediabetes awareness exploration (no intervention)	Culturally grounded awareness framing	Qualitative thematic analysis	Low prediabetes awareness; fatalistic beliefs	Awareness-focused insights to inform recruitment messaging	Reach
Joseph (1), 2023	Narrative review	Minority populations (national)	US health system/policy context	Diabetes prevention, treatment, and outcomes equity review	Equity-focused framing across diabetes care continuum	Translational/narrative synthesis	Broad scope limits specificity	Equity framing applicable across implementation domains	Adoption
Kim et al (18), 2018	Study protocol	Medicaid population	Digital diabetes prevention program (Medicaid)	Digital DPP adapted for Medicaid population	Adaptation for low-income/Medicaid-insured population	Nonrandomized comparative analysis	Selection bias; nonrandomized design	Real-world implementation context for underserved population	Implementation
Koh (19), 2023	Integrative review	Hispanic/Latino adults	Community-based nutrition program	Plant-based nutrition intervention	Culturally tailored plant-based dietary adaptation	Pre/post outcome analysis	Reliance on dietary self-report	Nutrition-focused cultural tailoring	Effectiveness
Ling et al (20), 2023	Narrative review	Minority adults (diabetes prevention)	Community-based peer support program	Peer-support intervention for diabetes prevention	Peer-led culturally relevant support model	Synthesis/comparative analysis	Limited Hispanic-specific data	Peer support enhances engagement and retention	Implementation
Loya et al (21), 2025	Pilot feasibility	Hispanic adults	Community-based physical activity program	Culturally tailored physical activity intervention	Culturally adapted physical activity programming	Pilot/feasibility pre/post analysis	Small sample size	Physical activity focus shows feasibility and acceptability	Effectiveness
McCurley et al (22), 2017	Pilot trial	Latina women	Primary care + community (Nuevo Amanecer-II)	Nuevo Amanecer-II culturally appropriate DPP	Promotora-led, Spanish-language, family-involved adaptation	Pilot pre/post analysis	Small sample size	Cultural relevance and promotora delivery model	Effectiveness
McCurley et al (8), 2017	Systematic review	Hispanic adults	Multiple community/clinical settings	Synthesis of culturally tailored DPP interventions	Synthesis of multiple cultural adaptation strategies	Systematic synthesis/narrative review	Not implementation-focused	Strong synthesis of effective adaptation strategies	Effectiveness
McEwen et al (23), 2010	Intervention trial	US–Mexico border populations	Border community health setting	Type 2 diabetes self-management social support intervention	Border-region, family/peer social support model	Pre/post intervention analysis	Older program model; limited generalizability	Social support drives sustained behavior change	Effectiveness
Meulenberg et al (24), 2025	Survey	NDPP lifestyle coaches (Latino-serving)	NDPP delivery sites serving Latino participants	NDPP delivery (coach-reported barriers/facilitators)	Coach-identified cultural adaptation needs	Survey/descriptive analysis	Self-report bias; coach-identified resource barriers	Barrier analysis informs workforce/training needs	Adoption
Miranda et al (25), 2025	Qualitative	Mexican-origin men	Community settings	Nutrition/health discussion using cultural dichos	Use of culturally rooted sayings (dichos) for health messaging	Qualitative thematic analysis	Gender-specific findings limit generalizability	Cultural narratives (dichos) enhance engagement among men	Reach
Ockene et al (26), 2012	RCT	Latino community adults (Lawrence, MA)	Community-based (Lawrence Latino Diabetes Prevention Project)	Community-based DPP-adapted lifestyle intervention	Spanish-language, community-delivered cultural adaptation	RCT with objective outcome measures	Single-region sample limits generalizability	Randomized design with community-based delivery success	Effectiveness
Patel et al (27), 2025	Scoping review	Population-level (multilevel interventions)	Multilevel settings	Multilevel diabetes prevention interventions	Multilevel cultural and contextual adaptation	Scoping/mixed-methods synthesis	Heterogeneous data across contexts	Multilevel lens applicable to adoption strategies	Adoption
Risica et al (28), 2018	Study protocol	Latino adults	Community clinic-based program	Vida Saludable/Healthy Life lifestyle change program	Community clinic–based cultural tailoring	Pilot methods/protocol-level analysis	Outcomes pending at time of publication	Clear program rationale and clinic-based delivery model	Implementation
Rosas et al (29), 2016	Study protocol: RCT	Primary care Latino adults	Primary care setting (Vida Sana)	Culturally adapted lifestyle intervention (Vida Sana)	Culturally adapted lifestyle program for cardiometabolic risk	RCT with rigorous outcome evaluation	Clinic-based delivery only	Rigorous design demonstrates effectiveness in primary care	Effectiveness
Rosas et al (30), 2018	Study protocol: RCT	Latino men (HOMBRE trial)	Community/clinical settings	HOMBRE intervention for obesity and prediabetes	Male-targeted culturally tailored intervention	RCT outcome analysis	Gender-specific design limits broader generalizability	Male engagement model addresses underrepresentation	Effectiveness
Rosas et al (31), 2018	Program description	Latino adults	Patient-centered care setting (Vida Sana)	Patient-centered diabetes prevention and weight loss approach	Patient-centered cultural tailoring	Patient-centered outcome analysis	Limited follow-up duration	Strong patient engagement and acceptability	Adoption
Rosas et al (32), 2024	Study protocol: RCT	Latina women (Vida Sana y Completa)	Primary care setting	Vida Sana y Completa culturally adapted DPP with grocery component	Innovative grocery/food-access cultural adaptation	RCT protocol-based outcome analysis	Resource-intensive intervention model	Innovative grocery-based component supports adherence	Effectiveness
Sanchez et al (33), 2022	Qualitative	Latinx families	Community/family settings	Healthy living facilitators/barriers exploration	Family-centered cultural framing (familismo)	Qualitative thematic analysis	Regional sample limits generalizability	Family dynamics identified as key facilitator	Reach
Saju et al (34), 2022	Qualitative	Diverse adults (including Hispanic/Latino) in lifestyle program	Community-based lifestyle intervention sites	Lifestyle intervention weight-loss program	Equity-focused exploration of race/ethnicity differences	Qualitative/comparative analysis	Recall bias in self-reported data	Equity focus highlights differential outcomes by group	Reach
Soltero et al (35), 2019	Multilevel intervention	Adults of Mexican descent (Maryvale community)	Multisector community settings (Viva Maryvale)	Community- and multi-level physical activity intervention	Multi-level, multi-sector cultural and environmental adaptation	Multilevel program evaluation	Complex model difficult to replicate	Multisector model demonstrates strong adoption pathway	Adoption
Vincent et al (36), 2007	Feasibility	Mexican Americans	Community-based setting	Early culturally tailored diabetes prevention intervention	Early-stage cultural tailoring for Mexican American adults	Feasibility analysis	Dated context; limited modern applicability	Early cultural tailoring established foundational model	Implementation
Vincent et al (37), 2013	Mixed methods	Adults of Mexican descent	Community-based program	Culturally tailored diabetes prevention program (recruitment/retention focus)	Recruitment/retention-focused cultural strategies	Mixed-methods recruitment/retention analysis	Small sample size	Recruitment and retention strategies identified	Reach, Implementation
Vincent et al (38), 2014	Quasi-experimental	Community-based adults of Mexican descent	Community-based setting	Community-based culturally tailored diabetes prevention intervention	Community-based cultural tailoring for high-risk adults	Quasi-experimental pre/post analysis	No randomization	Cultural tailoring associated with positive outcomes	Effectiveness
Joachim-Célestin et al (39), 2020	Qualitative	Latina participants (DPP enrollees)	Diabetes prevention program setting	Diabetes prevention program (participant perspective)	Exploration of Latina cultural perspectives on DPP enrollment	Qualitative thematic analysis	Subjective, self-reported data	Cultural insight into participant experience and motivation	Reach, Implementation
Lewis et al (40), 2025	Longitudinal qualitative	Vida Sana participants	Primary care/community setting	Vida Sana culturally adapted lifestyle program evaluated via RE-AIM framework	Long-term cultural adaptation sustainability strategies	Longitudinal qualitative analysis	Qualitative-only data limits generalizability	Sustainability insight from long-term participant follow-up	Maintenance

Abbreviations: CBPR, community-based participatory research; DPP, diabetes prevention program; NDPP, National Diabetes Prevention Program; RCT, randomized controlled trial; RE-AIM, Reach, Effectiveness, Adoption, Implementation, and Maintenance.

### Population characteristics

Mexican Americans were the most frequently represented subgroup across studies, followed by Puerto Ricans. Many studies reported participants as “Hispanic/Latino” without specifying their ethnic subgroup. Female participation averaged 78% (range, 65%–100%), with only 3 studies specifically studying men (25,30). Male participation ranged from 7% to 35% in mixed-sex studies, below population parity, despite Hispanic men experiencing comparable diabetes rates (1). Studies successfully reached high-risk populations: 64% enrolled low-income participants, 50% enrolled uninsured or Medicaid-insured participants, and 73% enrolled Spanish-speaking participants (22,29,38).

Table 3 provides a summary of facilitators, barriers, and notable outcomes across all 5 RE-AIM domains.

**Table 3:** RE-AIM Domain Results Summary, Review on Implementation Processes, Barriers, and Facilitators of Culturally Tailored Diabetes Prevention Interventions in Hispanic Communities (N = 37), 2005–2025^a^

RE-AIM domain	No. of studies reporting	Key facilitators	Key barriers	Notable outcomes
Reach	18	Community-based recruitment via trusted venues (75%); promotora-led recruitment (52%); culturally tailored materials/messaging (68%); family-based, *familismo*-oriented recruitment (39%); CBPR approaches (25%)	Low prediabetes awareness and fatalism (n = 12, 32%); language/health literacy barriers (n = 17, 46%); work schedule conflicts (n = 19, 51%); transportation/childcare barriers (n = 14, 38%); immigration concerns (n = 7, 19%); diabetes stigma (n = 9, 24%); gender-specific barriers for men (n = 6, 16%); digital access barriers (n = 8, 22%)	Enrollment ranged from 18% to 67% of eligible people (median, 43%); community-based intensive engagement achieved 45% to 67% enrollment; NDPP completion was 35.5%, with lower completion among Hispanic participants
Effectiveness	36	Cultural concordance; multiple (vs single) cultural adaptation strategies; promotora-led delivery comparable to professional-led delivery; community-based settings matched or exceeded clinical settings	Real-world effect sizes were smaller than those reported in the original DPP efficacy trial; heterogeneity in outcome reporting across studies	Weight loss, 3.2 kg to 5.8 kg; BMI reduction, 1.1 kg/m^2^ to 2.3 kg/m^2^; waist circumference reduction, 3.4 cm to 7.1 cm; HbA_1C_ reduction, 0.2 to 0.6 percentage points; participant satisfaction, 85% to 95%; attending 9 or more of 16 NDPP sessions was associated with 2 to 3 times more weight loss
Adoption	27	Mission alignment with health equity (33%); existing community relationships (43%); leadership support (22%); bilingual/bicultural staff (26%); external technical assistance (19%); funding availability (41%)	Resource constraints/inadequate funding (37%); lack of bicultural workforce (27%); organizational readiness gaps (15%); competing priorities (19%); sustainability concerns related to time-limited grants (26%); adaptation–fidelity tensions associated with CDC recognition requirements (11%)	Community-based organizations were the most common adopters; other adopting settings were faith-based organizations (33%), primary care clinics/FQHCs (26%), workplaces (7%), and educational settings (4%). Programs were delivered by CHWs/promotoras (52%), professional health educators (26%), or hybrid promotora–professional teams (15%).
Implementation	Multiple (cross-domain)	Cultural concordance and bilingual/bicultural staff as trusted cultural brokers; community-based delivery settings; CBPR approaches supporting adaptation without compromising fidelity; flexible scheduling accommodating work, caregiving, and transportation constraints	Limited time-bound funding constrained staffing and continuity; bicultural personnel shortages impeded scalability; digital literacy gaps challenged remote delivery; insufficient leadership buy-in within healthcare systems	Promotora-led interventions achieved cultural acceptability and cost-effectiveness comparable to professional-led models; CBPR strengthened recruitment and adaptation
Maintenance	1 (Primary focus); several others contributing related insights	Integration within existing health care infrastructure; institutional ownership; reimbursement mechanisms (eg, NDPP alignment); workforce stability; family involvement, peer support, and ongoing group interaction	Only 18% of studies reported outcomes beyond 12 months; sustainability was undermined by reliance on stand-alone, grant-funded initiatives; few studies included cost-effectiveness analyses or formal sustainability plans	Programs integrated into primary care and community health organizations demonstrated greater continuity than externally funded stand-alone programs; NDPP reimbursement-aligned programs were more likely to be sustained

Abbreviations: BMI, body mass index; CBPR, community-based participatory research; CDC, Centers for Disease Control and Prevention; CHW, community health worker; FQHC, federally qualified health center; HbA_1c_, hemoglobin A_1c_; NDPP, National Diabetes Prevention Program; RE-AIM, Reach, Effectiveness, Adoption, Implementation, and Maintenance.

a Percentages are calculated on all 37 included sources; some sources contributed data to more than 1 domain.

### Reach: participation and representativeness

Of the studies, 18 provided data relevant to the reach outcomes. Among those reporting participation rates, enrollment ranged from 18% to 67% of eligible people, with a median of 43% (5,10,37). A large-scale NDPP implementation study encountered challenges: only 35.5% of enrolled participants completed the core requirements, with even lower completion rates among Hispanic participants (3). Rural Hispanic adults faced limited access due to fewer available sites, geographic distances, and transportation issues (10). A community-based program using intensive engagement strategies reported higher enrollment rates, ranging from 45% to 67%, despite serving smaller eligible populations (22).

**Reach facilitators.** Community-based recruitment through trusted venues (churches, community centers) proved most effective (75% of studies). Promotora-led recruitment leveraged community health workers’ shared cultural backgrounds and existing trust relationships (52% of studies), achieving higher enrollment than professional recruiters (22). CBPR approaches were commonly associated with improved cultural appropriateness (25% of studies). Culturally tailored recruitment materials featuring Spanish language, culturally relevant imagery, and messaging emphasizing family health resonated powerfully with Hispanic adults (68% of studies) (12). Family-based recruitment, leveraging *familismo* values and flexible, low-barrier approaches that accommodate work schedules and reduce administrative burden, improved participation (39% of studies) (13,15,33).

**Reach barriers. **Low prediabetes awareness and fatalistic beliefs about diabetes undermined prevention messages (n =12 studies, 32%), with patients unaware of their prediabetes status or unfamiliar with available prevention options (17,41). Language and health literacy barriers persisted even with Spanish-language offerings, as technical verbiage intimidated prospective participants (n = 17, 46%). Work schedule conflicts affected low-income hourly workers without flexible schedules (n = 19, 51%) (39). Transportation and childcare barriers prevented attendance, especially in rural areas (n = 14, 38%). Immigration concerns deterred participation among undocumented immigrants and mixed-status families (n = 7, 19%). Diabetes-related stigma affected enrollment decisions (n = 9, 24%). Sex-specific barriers for Hispanic men included programs that men felt were designed for women, work inflexibility, discomfort with group settings, and cultural expectations of masculine self-sufficiency (n = 6, 16%). Digital access barriers limited the use of technology-delivered interventions among low-income participants (n = 8, 22%).

### Effectiveness: real-world outcomes

Community-based interventions produced clinically meaningful weight loss averaging 3.2 kg to 5.8 kg, body mass index reductions of 1.1 kg/m^2^ to 2.3 kg/m^2^, reductions in waist circumference of 3.4 cm to 7.1 cm, and reductions in hemoglobin A_1C_ of 0.2 to 0.6 percentage points (26,40). These real-world effect sizes were smaller than those in the original Diabetes Prevention Program (DPP) efficacy trial but were clinically significant (34). Dietary and physical activity improvements were substantial (21), with participants reporting sustained adoption of healthier practices culturally adapted to Hispanic preferences, including remotely delivered plant-based culinary programs that demonstrated feasibility and cultural acceptability among Hispanic adults (19,42). Participant satisfaction ratings were high (85%–95%) (36).

**Implementation context effects. **A community-based setting achieved outcomes comparable to or exceeding clinical settings while achieving higher levels of engagement (26). A promotora-led intervention reached results comparable to professional-led interventions while improving cultural acceptability and cost-effectiveness (22). Per NDPP data, dose–response relationships underscored retention’s importance: participants completing 9 or more of 16 NDPP sessions achieved 2 to 3 times more weight loss than those attending fewer sessions (3). Interventions using multiple cultural adaptation strategies achieved superior outcomes compared with single-strategy adaptations (31,40).

### Adoption: organizational settings and delivery personnel

Twenty-seven studies provided data on organizational adoption. Community-based organizations were the most common adopters, offering trusted venues and cultural concordance, although resource constraints were typical. Faith-based organizations (33%) successfully adopted programs by leveraging social networks and trusted religious leaders but faced challenges with volunteer-dependent delivery. Primary care clinics and federally qualified health centers (26%) integrated programs but faced capacity constraints and competing priorities (11,28). Workplace (7%) and educational settings (4%) showed promise but faced sustainability challenges,

Delivery personnel varied across studies: community health workers/promotoras (52%) brought shared cultural backgrounds and community trust but faced recruitment challenges and limited career pathways; professional health educators (26%) provided clinical expertise but with limited bilingual/bicultural availability; hybrid models (15%), which partnered promotoras with professionals, optimized cultural responsiveness and clinical quality.

**Adoption facilitators.** Mission alignment with health equity (33% of studies), existing community relationships (43%), leadership support (22%), bilingual/bicultural staff characteristics (26%), external technical assistance (19%), and funding availability (41%) facilitated organizational adoption.

**Adoption barriers.** Resource constraints, including limited staff capacity and inadequate funding (37%), lack of a bicultural workforce (27%), organizational readiness gaps (15%), competing priorities (19%), sustainability concerns about time-limited grants (26%), and adaptation-fidelity tensions related to Centers for Disease Control and Prevention recognition requirements (11%), impeded adoption.

### Implementation

Implementation success across studies was linked to cultural concordance and to predominantly bilingual/bicultural staff serving as trusted cultural brokers (22). One program delivered in trusted community settings reduced structural barriers and increased acceptability (35). CBPR approaches strengthened recruitment and supported adaptation without compromising core components (31). Flexibility accommodating work schedules, caregiving responsibilities, and transportation constraints improved attendance and retention (14).

Implementation barriers identified across studies included limited time-bound funding, which constrained staffing and continuity; bicultural personnel shortages, which impeded scalability; digital literacy gaps, which challenged remotely delivered interventions (18); and insufficient leadership buy-in, which complicated implementation in health care systems (2,24).

### Maintenance

Maintenance was the least consistently examined domain, with only 1 analytical study (40) clearly treating it as a primary focus, although several others provided maintenance-related insights. Only 18% of studies reported outcomes beyond 12 months. Sustainability was closely linked to integration within existing health care infrastructures rather than to stand-alone, grant-funded initiatives (2). Programs integrated into primary care or community health organizations demonstrated greater continuity than those dependent on short-term external funding.

At the individual level, sustained behavior change was supported by family involvement, peer support, and ongoing group interaction (20,23). Institutional ownership, reimbursement mechanisms, and workforce stability facilitated organizational sustainability. Programs aligned with NDPP reimbursement pathways were more likely to persist (40). Few studies reported cost-effectiveness analyses or formal sustainability plans, limiting assessment of long-term feasibility (40).

## Discussion

This integrative review of 37 studies showed that culturally tailored diabetes prevention interventions for Hispanic adults are effective at reducing diabetes risk; however, their translation into sustained real-world practice is uneven. Applying the RE-AIM framework showed that, while reach and effectiveness are well documented, adoption, implementation, and maintenance lag, limiting population-level impact.

Interventions grounded in cultural concordance, community partnerships, and flexible delivery showed stronger implementation outcomes than clinic-based or technology-driven models alone (16,35). Promotora-led and family-centered approaches effectively fostered trust, relevance, and engagement, underscoring the importance of relational and culturally embedded strategies (43). However, persistent gaps, including male underrepresentation, workforce shortages, and sustainability limitations, underscore the need for intentional implementation planning that addresses structural barriers alongside intervention design.

The persistent gap between reach/effectiveness and the downstream domains of adoption, implementation, and maintenance reflects well-documented patterns in implementation science; interventions that demonstrate efficacy under controlled conditions frequently encounter structural, organizational, and resource barriers that impede real-world translation. Community-based organizations, while culturally aligned, frequently lack the infrastructure, reimbursement mechanisms, and workforce stability to sustain programming beyond initial grant periods, a phenomenon consistent with the implementation outcomes framework by Proctor et al, which identifies sustainability as the most distal and difficult-to-achieve implementation outcome (44).

Moreover, community partnerships improve implementation outcomes by reducing the structural distance between intervention design and the delivery context, facilitating real-time adaptation without compromising core components, a process that Glasgow et al describe as “fit” within the RE-AIM framework (7). Flexible delivery accommodates the lived realities of low-income, hourly-wage, and caregiving populations for whom rigid scheduling constitutes a participation barrier independent of intervention quality (2).

Promotora-led models are effective because promotoras serve as cultural and epistemological agents, people who share the participants’ language, cultural frameworks, and social contexts and who, therefore, communicate health information through culturally resonant meaning systems (22). Family-centered approaches leverage *familismo*, a core Hispanic cultural value emphasizing collective family identity and interdependence, thereby transforming behavior change from an individual responsibility into a shared family commitment, a reframing that reduces stigma and increases social accountability (44). The qualitative review by Sanders Thompson et al similarly identified culturally grounded theoretical frameworks as essential to intervention engagement and retention among Hispanic adults (43).

### Implications for public health practice

Translating evidence-based diabetes prevention into sustainable community practice requires strategic implementation that addresses multilevel barriers (27). Community-based organizations demonstrated strong cultural alignment and trust but faced resource constraints that threatened continuity. Health care and public health workforces are well positioned to operationalize culturally tailored prevention strategies through partnerships with trusted community organizations, including faith-based settings (45), flexible delivery models that reduce structural barriers to participation, and the integration of cultural humility and language concordance into service delivery.

Addressing upstream factors, including stigma, gender norms, health literacy, and social determinants, is essential to improving reach and engagement. Persistent underrepresentation of Hispanic men across studies underscores the need for targeted outreach, including workplace-based programs, male-specific messaging, and interventions that address cultural expectations of masculine self-sufficiency. Rural communities face compounded access barriers related to geographic distance, transportation, and limited program availability, necessitating deliberate strategies such as telehealth adaptation, mobile delivery, and regional coordination.

The success of promotora-led models underscores the value of leveraging community health workers’ shared cultural backgrounds, existing trust relationships, and community embeddedness. However, workforce development must address recruitment challenges, limited career pathways, and the need for ongoing training and support. Hybrid models that pair community health workers with professional health educators optimize both cultural responsiveness and clinical quality while expanding workforce capacity.

### Implications for policy and systems

Sustainable diabetes prevention requires structural support that extends beyond time-limited grant funding. Policy priorities include reimbursement for community health workers and promotoras, investment in bicultural workforce development, and incentives for health care–community partnerships (1,2). Without policy alignment, culturally tailored interventions risk remaining fragmented, short-term, and grant-dependent rather than embedded in health care and public health systems.

Integration into existing health care infrastructures demonstrated greater continuity than stand-alone initiatives. Programs aligned with NDPP reimbursement pathways were more likely to persist (40). Policy support for prevention services, workforce development, and health care–community integration is essential to achieving equitable access and sustained impact. Policies that address social determinants of health, which include immigration concerns, transportation barriers, childcare access, and inflexible work schedules, can reduce structural barriers that limit participation among populations at high risk of poor health outcomes.

### Implications for the workforce

Implementation success depends on a culturally concordant, well-trained, and sustainably employed workforce. Findings from studies underline the need to equip public health and health care professionals with competencies in cultural adaptation, community-based participatory approaches, and implementation science frameworks. Educational programs should emphasize partnership development, health equity advocacy, and systems thinking to address multilevel barriers to diabetes prevention.

Public health workers, particularly those in community and public health settings, play essential roles in bridging evidence, culture, and community context. Their population-focused lens helps identify groups requiring targeted outreach, and their competencies in cultural humility, trust-building, and addressing social determinants position them to lead implementation efforts. However, successful workforce development extends beyond individual competencies to include career pathways for community health workers, continuing education and technical assistance, and organizational support for implementation fidelity and adaptation (2).

Integrating RE-AIM and similar implementation frameworks into workforce development strengthens the capacity to translate evidence-based interventions into equitable, sustainable community practice. Workforce planning must address bilingual/bicultural personnel shortages, which have emerged as a persistent implementation barrier to scalability and adoption across diverse settings (24).

### Recommendations for future research

Research priorities that emerged from the synthesis of 37 studies include comparative implementation trials that identify which strategies work best in specific contexts (45), long-term maintenance studies extending beyond 12 months, cost-effectiveness analyses to inform sustainability decisions, tailored strategies to engage Hispanic men and underrepresented subgroups, and hybrid effectiveness–implementation designs that advance equitable, scalable prevention (27,40). 

Inconsistent reporting of implementation outcomes, predominantly in the adoption and maintenance domains, limits evidence synthesis and cross-study comparisons. Future research should systematically measure and report RE-AIM outcomes using standardized metrics to strengthen the evidence base for implementation decision-making. Research examining workforce contributions to diabetes prevention implementation would inform workforce planning and educational preparation.

### Limitations

Although comprehensive, the review had several limitations. These articles were limited to English-language studies conducted in the US, potentially excluding relevant international perspectives. The variability in study designs, intervention components, and outcome reporting across the 37 studies limited direct comparisons. Implementation outcomes were inconsistently reported, particularly in the adoption and maintenance domains, with only 2 studies examining maintenance beyond 12 months. The inclusion of study protocols and reviews alongside empirical studies broadened the implementation evidence but means that not all sources contributed outcome data; effectiveness findings are drawn from the empirical studies only.

Despite an equity focus, some studies may have excluded structurally marginalized people, including people who are undocumented or those with unstable housing. Persistent male underrepresentation limits generalizability to Hispanic men. Furthermore, quality appraisal and data extraction were conducted by a single reviewer, which is a methodological limitation. Although standardized Johns Hopkins Evidence-Based Practice criteria were applied consistently and the reviewer has expertise in diabetes and implementation science, the absence of a second independent appraiser increases the risk of bias and limits the confirmability of quality ratings. Future reviews should incorporate dual reviewers and inter-rater reliability calculations to strengthen methodological rigor.

### Conclusion

This integrative review demonstrates that culturally tailored diabetes prevention interventions for Hispanic adults are effective, acceptable, and culturally resonant. Yet, their real-world impact is limited by implementation and sustainability challenges. The application of the RE-AIM framework to analyze 37 primary research studies indicated gaps beyond effectiveness, particularly in adoption, implementation, and maintenance, which hinders equitable benefits at the population level.

Evidence from studies supports cultural concordance, community-based delivery, promotora models, and family-centered approaches as foundational to successful diabetes prevention in Hispanic populations. However, persistent barriers that include workforce limitations, funding instability, male underrepresentation, and limited maintenance data highlight the need for intentional implementation planning and sustainability-focused research.

Advancing equitable diabetes prevention requires coordinated efforts across practice, education, research, and policy to ensure that interventions are culturally tailored, accessible, scalable, and sustainable. Addressing structural inequities through workforce development, sustainable funding mechanisms, and health care–community partnerships is essential to translating evidence into population-level impact.

## References

[1] Joseph JJ . Advancing equity in diabetes prevention, treatment, and outcomes. *Endocrinol Metab Clin North Am.* 2023;52(4):559–572. 10.1016/j.ecl.2023.05.001 37865473

[2] Burd C , Gruss S , Albright A , Zina A , Schumacher P , Alley D . Translating knowledge into action to prevent type 2 diabetes: Medicare expansion of the National Diabetes Prevention Program lifestyle intervention. *Milbank Q.* 2020;98(1):172–196. 10.1111/1468-0009.12443 31994260 PMC7077780

[3] Cannon MJ , Masalovich S , Ng BP , Soler RE , Jabrah R , Ely EK , . Retention among participants in the National Diabetes Prevention Program lifestyle change program, 2012–2017. *Diabetes Care.* 2020;43(9):2042–2049. 10.2337/dc19-2366 32616617 PMC11000538

[4] Gutierrez Chavez M , Parsons H , Gutner CA , Asnaani A , Baucom KJW . Investigating the delivery of the National Diabetes Prevention Program to Latines: coaches’ perspectives. *Transl Behav Med.* 2025;15(1):ibaf024. 10.1093/tbm/ibaf024 40539602 PMC12204697

[5] Johnston CE , MacPherson MM , Jung ME . Are diabetes prevention programs reaching those most at risk? A scoping review. *Can J Diabetes.* 2025;49(2):104–113.e1. 10.1016/j.jcjd.2024.11.006 39617265

[6] Dineen TE , Bean C , Jung ME . Implementation of a diabetes prevention program within two community sites: a qualitative assessment. *Implement Sci Commun.* 2022;3(1):11. 10.1186/s43058-022-00258-6 35123582 PMC8817168

[7] Glasgow RE , Harden SM , Gaglio B , Rabin B , Smith ML , Porter GC , . RE-AIM planning and evaluation framework: adapting to new science and practice with a 20-year review. *Front Public Health.* 2019;7:64. 10.3389/fpubh.2019.00064 30984733 PMC6450067

[8] McCurley JL , Gutierrez AP , Gallo LC . Diabetes prevention in U.S. Hispanic adults: a systematic review of culturally tailored interventions. *Am J Prev Med.* 2017;52(4):519–529. 10.1016/j.amepre.2016.10.028 27989451 PMC5362335

[9] Whittemore R , Knafl K . The integrative review: updated methodology. *J Adv Nurs.* 2005;52(5):546–553. 10.1111/j.1365-2648.2005.03621.x 16268861

[10] Ariel-Donges AH , Gordon EL , Dixon BN , Eastman AJ , Bauman V , Ross KM , . Rural/urban disparities in access to the National Diabetes Prevention Program. *Transl Behav Med.* 2020;10(6):1554–1558. 31228199 10.1093/tbm/ibz098 PMC7796706

[11] Balan A , Gugerty MC , Shreve K , Ayyagari R , Brady M , Williams J , . Advancing equity in diabetes prevention for both Black and Hispanic women: an executive summary of lessons learned and action steps. *AJPM Focus.* 2025;4(2):100314. 10.1016/j.focus.2025.100314 40124257 PMC11928944

[12] Brown SA , Perkison WB , García AA , Cuevas HE , Velasquez MM , Winter MA , . The Starr County Border Health Initiative: focus groups on diabetes prevention in Mexican Americans. *Diabetes Educ.* 2018;44(3):293–306. 10.1177/0145721718770143 29644932 PMC6349423

[13] Cranston KD , MacPherson MM , Sim JAP , Jung ME . Small steps towards an inclusive diabetes prevention program: how small steps for big changes is improving program equity and inclusion. *Community Health Equity Res Policy.* 2025;45(3):227–236. 10.1177/2752535X231189932 37478867

[14] Fontil V , McDermott K , Tieu L , Rios C , Gibson E , Castro Sweet C , . Adaptation and feasibility study of a digital health program to prevent diabetes among low-income patients: results from a partnership between a digital health company and an academic research team. *J Diabetes Res.* 2016;2016:8472391. 10.1155/2016/8472391 27868070 PMC5102733

[15] Franklin BE , Meulenberg BL , Beaulieu EZ , Cisneros-Macias J , Cao Y , Carbajal-Salisbury S , . “PrevenganT2,” a culturally responsive family-based diabetes prevention intervention for Hispanic or Latino adults at high risk for type 2 diabetes. *JMIR Res Protoc.* 2025;14:e66317. 10.2196/66317 40811792 PMC12395107

[16] Horowitz CR , Eckhardt S , Talavera S , Goytia C , Lorig K . Effectively translating diabetes prevention: a successful model in a historically underserved community. *Transl Behav Med.* 2011;1(3):443–452. 10.1007/s13142-011-0067-6 22189897 PMC3242468

[17] Joiner KL , Adams M , Minehan EB , O’Brien MJ . Perceptions and experiences of developing prediabetes awareness and participating in the National Diabetes Prevention Program among Hispanic US adults: qualitative study. *JMIR Form Res.* 2025;9:e66964. 10.2196/66964 39945459 PMC11835594

[18] Kim SE , Castro Sweet CM , Gibson E , Madero EN , Rubino B , Morrison J , . Evaluation of a digital diabetes prevention program adapted for the Medicaid population: study design and methods for a non-randomized, controlled trial. *Contemp Clin Trials Commun.* 2018;10:161–168. 10.1016/j.conctc.2018.05.007 30035241 PMC6052649

[19] Koh LM . Culturally tailoring plant-based nutrition interventions for Hispanic/Latino adults at risk for or with type 2 diabetes: an integrative review. *Hisp Health Care Int.* 2023;21(2):89–103. 10.1177/15404153221085696 35257611 PMC11600662

[20] Ling I , Zambrana RE , Echeverria S , López L . Peer support to enhance type 2 diabetes prevention among African American and Latino adults. *Endocrinol Metab Clin North Am.* 2023;52(4):573–583. 10.1016/j.ecl.2023.05.012 37865474

[21] Loya J , Garcia DO , Maldonado A , Villavicencio E . A culturally tailored physical activity intervention for Hispanic adults living with type 2 diabetes: pre–post pilot feasibility study. *JMIR Diabetes.* 2025;10:e62876. 10.2196/62876 40493665 PMC12173091

[22] McCurley JL , Fortmann AL , Gutierrez AP , Gonzalez P , Euyoque J , Clark T , . Pilot test of a culturally appropriate diabetes prevention intervention for at-risk Latinos: Nuevo Amanecer-II. *Diabetes Educ.* 2017;43(6):631–640. 10.1177/0145721717738020 29059040 PMC5849058

[23] McEwen MM , Pasvogel A , Gallegos G , Barrera L . Type 2 diabetes self-management social support intervention at the U.S.–Mexico border. *Public Health Nurs.* 2010;27(4):310–319. 10.1111/j.1525-1446.2010.00860.x 20626831 PMC2917267

[24] Meulenberg BL , Kim T , Diaz JE , Gutierrez Chavez M , Asnaani A , Gutner C , . Lifestyle coach perceptions of barriers and facilitators to participation and lifestyle change among Black and Latinx National Diabetes Prevention Program participants. *Am J Health Educ.* 2025;56(5):423–437. 10.1080/19325037.2024.2421541 41019360 PMC12463402

[25] Miranda A , Sánchez C , Garcia DO , Gonzalez L , Guevara D , Garcia P , . Deciphering dichos for nutrition and health discussion: a qualitative study among men of Mexican origin with or at-risk of type 2 diabetes. *J Nutr Educ Behav.* 2025;57(1):18–28. 10.1016/j.jneb.2024.09.003 39425723

[26] Ockene IS , Tellez TL , Rosal MC , Reed GW , Mordes J , Merriam PA , . Outcomes of a Latino community-based intervention for the prevention of diabetes: the Lawrence Latino Diabetes Prevention Project. *Am J Public Health.* 2012;102(2):336–342. 10.2105/AJPH.2011.300357 22390448 PMC3483988

[27] Patel R , Kornas K , Gerstle D , Diemert LM , Rosella LC . Multilevel diabetes prevention interventions to address population inequities in diabetes risk: scoping review. *JMIR Public Health Surveill.* 2025;11:e70267. 10.2196/70267 40854300 PMC12377877

[28] Risica PM , McCarthy M , Barry K , Oliverio SP , De Groot AS . Community clinic-based lifestyle change for prevention of metabolic syndrome: rationale, design and methods of the ‘Vida Sana/Healthy Life’ program. *Contemp Clin Trials Commun.* 2018;12:123–128. 10.1016/j.conctc.2018.10.002 30417157 PMC6218840

[29] Rosas LG , Lv N , Xiao L , Lewis MA , Zavella P , Kramer MK , . Evaluation of a culturally-adapted lifestyle intervention to treat elevated cardiometabolic risk of Latino adults in primary care (Vida Sana): a randomized controlled trial. *Contemp Clin Trials.* 2016;48:30–40. 10.1016/j.cct.2016.03.003 26995280 PMC4886337

[30] Rosas LG , Lv N , Azar KM , Xiao L , Hooker SP , Lewis MA , . HOMBRE: a randomized controlled trial to compare two approaches to weight loss for overweight and obese Latino men. *Contemp Clin Trials.* 2018;68:23–34. 10.1016/j.cct.2018.02.019 29505867

[31] Rosas LG , Lv N , Lewis MA , Venditti EM , Zavella P , Luna V , . A Latino patient-centered, evidence-based approach to diabetes prevention. *J Am Board Fam Med.* 2018;31(3):364–374. 10.3122/jabfm.2018.03.170280 29743220

[32] Rosas LG , Perez JA , Chen W-T , Xiao L , Rodriguez Espinosa P , Venditti EM , . Vida Sana y Completa: a randomized controlled trial to examine the effectiveness of diabetes prevention with and without medically supportive groceries among Latina women. *Contemp Clin Trials.* 2024;143:107582. 10.1016/j.cct.2024.107582 38810932 PMC12499523

[33] Sanchez N , Bernstein R , Annameier SK , Clark ELM , Jimenez V , Hanson L , . Identification of facilitators and barriers of healthy living and type 2 diabetes prevention among Latinx families. *J Lat Psychol.* 2022;10(3):225–240. 10.1037/lat0000205 36211633 PMC9536431

[34] Saju R , Castellon-Lopez Y , Turk N , Moin T , Mangione CM , Norris KC , . Differences in weight loss by race and ethnicity in the PRIDE trial: a qualitative analysis of participant perspectives. *J Gen Intern Med.* 2022;37(14):3715–3722. 10.1007/s11606-022-07521-5 35469358 PMC9037581

[35] Soltero EG , Ramos C , Williams AN , Hooker E , Mendez J , Wildy H , . ¡Viva Maryvale!: a multilevel, multisector model to community-based diabetes prevention. *Am J Prev Med.* 2019;56(1):58–65. 10.1016/j.amepre.2018.07.034 30573148 PMC6815673

[36] Vincent D , Pasvogel A , Barrera L . A feasibility study of a culturally tailored diabetes intervention for Mexican Americans. *Biol Res Nurs.* 2007;9(2):130–141. 10.1177/1099800407304980 17909165

[37] Vincent D , McEwen MM , Hepworth JT , Stump CS . Challenges and success of recruiting and retention for a culturally tailored diabetes prevention program for adults of Mexican descent. *Diabetes Educ.* 2013;39(2):222–230. 10.1177/0145721713475842 23439692 PMC6383721

[38] Vincent D , McEwen MM , Hepworth JT , Stump CS . The effects of a community-based, culturally tailored diabetes prevention intervention for high-risk adults of Mexican descent. *Diabetes Educ.* 2014;40(2):202–213. 10.1177/0145721714521020 24510942 PMC6383713

[39] Joachim-Célestin M , Gamboa-Maldonado T , Dos Santos H , Montgomery SB . A qualitative study on the perspectives of Latinas enrolled in a diabetes prevention program: is the cost of prevention too high? *J Prim Care Community Health.* 2020;11:2150132720945423. 10.1177/2150132720945423 32755275 PMC7543125

[40] Lewis MA , Wagner LK , Rosas LG , Lv N , Venditti EM , Zavella P , . Examining the potential public health impact of Vida Sana using the RE-AIM framework: a longitudinal qualitative study. *Transl Behav Med.* 2025;15(1):ibaf067. 10.1093/tbm/ibaf067 41231199 PMC12613243

[41] O’Brien MJ , Moran MR , Tang JW , Vargas MC , Talen M , Zimmermann LJ , . Patient perceptions about prediabetes and preferences for diabetes prevention. *Diabetes Educ.* 2016;42(6):667–677. 10.1177/0145721716666678 27621093 PMC5228412

[42] Koh LM , Iradukunda F , Martínez AD , Caetano Schulz KC , Bielitz I , Walker RK . A remotely accessible plant-based culinary intervention for Latina/o/x adults at risk for diabetes: lessons learned. *Front Nutr.* 2024;11:1298755. 10.3389/fnut.2024.1298755 38414490 PMC10896850

[43] Sanders Thompson VL , Johnson-Jennings M , Bauman AA , Proctor E . Use of culturally focused theoretical frameworks for adapting diabetes prevention programs: a qualitative review. *Prev Chronic Dis.* 2015;12:E60. 10.5888/pcd12.140421 25950567 PMC4436044

[44] Proctor E , Silmere H , Raghavan R , Hovmand P , Aarons G , Bunger A , . Outcomes for implementation research: conceptual distinctions, measurement challenges, and research agenda. *Adm Policy Ment Health.* 2011;38(2):65–76. 10.1007/s10488-010-0319-7 20957426 PMC3068522

[45] McElfish PA , Long CR , Kaholokula JK , Aitaoto N , Bursac Z , Capelle L , . Design of a comparative effectiveness randomized controlled trial testing a faith-based Diabetes Prevention Program (WORD DPP) vs. a Pacific culturally adapted Diabetes Prevention Program (PILI DPP) for Marshallese in the United States. *Medicine (Baltimore).* 2018;97(19):e0677. 10.1097/MD.0000000000010677 29742712 PMC5959435

